# IRF1 is critical for the TNF-driven interferon response in rheumatoid fibroblast-like synoviocytes: JAKinibs suppress the interferon response in RA-FLSs

**DOI:** 10.1038/s12276-019-0267-6

**Published:** 2019-07-08

**Authors:** Michael Bonelli, Karolina Dalwigk, Alexander Platzer, Isabel Olmos Calvo, Silvia Hayer, Birgit Niederreiter, Johannes Holinka, Florian Sevelda, Thomas Pap, Günter Steiner, Giulio Superti-Furga, Josef S. Smolen, Hans P. Kiener, Thomas Karonitsch

**Affiliations:** 1 Division of Rheumatology, Department of Medicine 3, Medical University of Vienna, 1090 Vienna, Austria; 2 Department of Orthopaedics, Medical University of Vienna, 1090 Vienna, Austria; 3 Institute of Musculoskeletal Medicine, University Hospital Muenster, 48149 Muenster, Germany; 4 Ludwig Boltzmann Institute for Arthritis and Rehabilitation, Vienna, Austria; 5 CeMM Research Center for Molecular Medicine of the Austrian Academy of Sciences, 1090 Vienna, Austria

**Keywords:** Chronic inflammation, Interferons

## Abstract

Rheumatoid arthritis (RA) is an autoimmune disease characterized by persistent synovial inflammation. The major drivers of synovial inflammation are cytokines and chemokines. Among these molecules, TNF activates fibroblast-like synoviocytes (FLSs), which leads to the production of inflammatory mediators. Here, we show that TNF regulates the expression of the transcription factor interferon regulatory factor 1 (IRF1) in human FLSs as well as in a TNF transgenic arthritis mouse model. Transcriptomic analyses of IRF1-deficient, TNF-stimulated FLSs define the interferon (IFN) pathway as a major target of IRF1. IRF1 expression is associated with the expression of IFNβ, which leads to the activation of the JAK-STAT pathway. Blocking the JAK-STAT pathway with the Janus kinase inhibitor (JAKinib) baricitinib or tofacitinib reduces the expression of IFN-regulated genes (IRGs) in TNF-activated FLSs. Therefore, we conclude that TNF induces a distinct inflammatory cascade, in which IRGs are key elements, in FLSs. The IFN-signature might be a promising biomarker for the efficient and personalized use of new treatment strategies for RA, such as JAKinibs.

## IRF-1: a key regulator in rheumatoid Arthritis

Thomas Karonitsch at the Medical University of Vienna, Austria, and colleagues showed that activation of the protein interferon regulatory factor 1 (IRF-1) in human synoviocytes promoted the expression of genes that contribute to sustained synovial inflammation in rheumatoid Arthritis (RA). They found that the Januse kinase (JAK) inhibitors baricitinb and tofacitinib, which are both approved for the treatment of RA, prevented the activation of IRF1 regulated proinflammatory genes. Their findings unvover a potential role for IRF1 activated genes as a biomarker to predict the treatment response to RA therapies and moreover highlight the benefit of targeting inflammatory pathways in synoviocytes for the treatment of RA.

## Introduction

Rheumatoid arthritis (RA) is a chronic inflammatory autoimmune disease that primarily affects the synovium of diarthrodial joints. Rheumatoid synovitis is characterized by the expansion of resident fibroblast-like synoviocytes (FLSs) and infiltration of immune cells into the synovial membrane^1,2^. Cytokine- and chemokine-mediated crosstalk between activated immune cells and FLSs sustains local inflammation and imprints disease-specific cellular signatures^3^. Whole-genome expression profiling of rheumatoid synovial tissue samples depicts a potential role for interferon-regulated genes (IRGs) in the pathogenesis of RA. Among the IRGs, several genes that drive RA disease progression, such as the transcription factor (TF) *STAT1*, the chemokines *CXCL9* and *CXCL10*, and the cytokine *TNFSF13B*, which is also called B-cell activating factor (BAFF), can be found^4,5^. The overexpression of IRGs can be detected in up to 65% of RA patients^6^. Individuals with an interferon (IFN) signature have a significantly higher risk of developing RA^7^, underlining the major role of IRGs in the pathogenesis of RA. The IFN signature has also been discussed as a potential biomarker, since clinical studies have demonstrated strong associations between IRGs and the clinical response to biological disease modifying antirheumatic drugs (bDMARDS)^8–11^. In RA patients, it has been shown that TNF is a strong inducer of IRGs^12^. Especially in FLSs, we previously demonstrated that the TNF-induced signature contains more than 50% of the IRGs^13^. However, the signaling circuit that allows TNF to induce the expression of IRGs in FLSs is largely unknown.

Previous studies highlighted a key role for the TF IRF1 in TNF-induced gene expression in monocytes and endothelial cells^14,15^. Since IRF1 knockdown prevents murine collagen-induced arthritis^16^, and IRF1 is expressed in activated FLSs^17^, we hypothesized that IRF1 might contribute to the TNF response in RA-FLSs. Through a comprehensive approach encompassing transcriptomics and biochemistry, our study indeed revealed that IRF1 specifically drives the IFN signature in RA-FLSs via STAT1. Accordingly, blocking the JAK-STAT pathway with a JAK inhibitor (JAKinib), baricitinib or tofacitinib, suppressed the TNF-induced expression of proinflammatory IRGs. Thus, our study contributes novel insights into the synovial response to TNF and shows that JAKinibs target the TNF-induced IFN response in RA.

## Material and methods

### Patients and synovial tissue samples

Synovial tissue samples were obtained from RA (fulfilling the American College of Rheumatology/European League Against Rheumatism (ACR/EULAR) classification criteria for RA^18^) or osteoarthritis (OA) patients undergoing joint replacement or synovectomy. All patients provided written informed consent prior to synovial tissue donation. This study was approved by the ethics committee of the Medical University of Vienna.

### hTNFtg arthritis mouse model

TNF transgenic mice that overexpress human TNF (Tg197 strain, C57BL/6 genetic background) were originally generated by the group of George Kollias (Fleming Institute, Athens, Greece^19^). Mice were maintained under conventional housing conditions (humidity 50%, 22 °C, 12-h light/12-h dark cycle). All experiments were performed with female mice. Age-matched nontransgenic female littermates were used as controls. All experiments were approved by the local ethical committee and the Federal Ministry of Science, Research and Economics.

### Immunohistochemistry (IHC) of synovial tissue samples

Synovial tissue samples (patient characteristics are shown in Supplementary Table 1) were fixed in paraformaldehyde and then embedded in paraffin. Paraffin-embedded sections were treated with Tris-EDTA (pH 9). To reduce nonspecific protein binding, the sections were incubated with goat serum. Synovial IRF1 expression was detected with a polyclonal rabbit anti-IRF1 antibody (Cell Signaling Technology). A nonimmune immunoglobulin of the same isotype and concentration as the primary antibody (anti-rabbit IgG (R&D Systems)) served as a control. After incubation with a biotinylated goat anti-rabbit antibody (Vector), the sections were incubated with Vectastain Elite reagent and visualized using 3,3-diaminobenzidine (Vector). The sections were counterstained with hematoxylin (Merck). The expression of IRF1 was assessed using semiquantitative scoring (0 = no staining, 3 = high staining).

IHC was also performed on hind paw tissue from 15-week-old hTNFtg mice and wild-type (WT) littermates. Hind paws were fixed in 7% formaldehyde for 6 h, followed by decalcification in 14% EDTA buffer (pH 7.2) for 4–6 days. Paraffin-embedded sections were used for immunohistochemical staining for synovial IRF1 expression using the antibodies and protocol mentioned above.

### Isolation and culture of FLSs

FLS single cell suspensions were obtained by digesting minced synovial tissue samples with collagenase type II (Merck). FLSs were cultured in DMEM (Thermo Fisher Scientific) supplemented with 10% fetal bovine serum (FBS; HyClone), 1% penicillin/streptomycin (P/S), and nonessential amino acids (both Thermo Fisher Scientific). FLSs beyond passage 4 were used. The following cytokines and inhibitors were used as indicated: TNF (10 ng/ml, R&D Systems), baricitinib (250 nM, Selleckchem) and tofacitinib (250 nM, Selleckchem).

### Western blot analysis

FLSs were lysed with RIPA buffer (Thermo Fisher Scientific) supplemented with the Halt™ phosphatase inhibitor cocktail (Thermo Fisher Scientific) and a protease inhibitor mix (Sigma-Aldrich). The protein lysates were fractioned on polyacrylamide gels, followed by electrotransfer to nitrocellulose membranes, which were blocked with either 5% BSA or 5% nonfat dry milk and then incubated with primary antibodies (Cell Signaling Technology: anti-IκBα, anti-IRF1, anti-BAFF, anti-p-STAT1, and anti-STAT1; Sigma-Aldrich: anti-ACTIN). After an incubation with HRP-conjugated secondary antibodies (Cell Signaling Technology), specific bands were detected with the BIORAD Clarity ECL Western substrate. Reprobing was performed using ReBlot Plus Strong Solution (Merck).

### Synovial micromass cultures

Micromass organ cultures were prepared as previously described^20^. FLSs were resuspended in ice-cold Matrigel Matrix (BD Biosciences). The cell/ECM suspension was placed on Poly-HEMA-coated culture dishes (Sigma-Aldrich) and overlaid with culture medium (DMEM supplemented with 5% FBS, 1% ITS liquid media supplement (Sigma-Aldrich), 0.125% bovine serum albumin (BSA; Calbiochem), 0.008 g ascorbic acid (Thermo Fisher Scientific), 1% NEAA (Thermo Fisher Scientific) and 1% P/S). At the time points indicated, the micromasses were fixed in paraformaldehyde and then embedded in paraffin. Paraffin-embedded sections were treated with a citrate buffer (pH 6, BAFF) or Tris-EDTA (pH 9, IRF1). To reduce nonspecific protein binding, the sections were incubated with the goat serum. The following antibodies were used: anti-IRF1 (Cell Signaling Technology) and anti-BAFF (Enzo Life Sciences). Nonimmune immunoglobulins of the same isotype and concentration as the primary antibody (anti-rabbit IgG, Novus Biologicals; rat IgM, Thermo Fisher Scientific) served as controls.

### siRNA-mediated expression knockdown

FLSs were cultured in Opti-MEM (Thermo Fisher Scientific) and transfected with SMARTpool: ON-TARGETplus siRNA pools (Horizon Discovery) using Lipofectamine (Thermo Fisher Scientific)^21^.

### RNA isolation

RNA was isolated from RA-FLSs using an RNeasy purification kit (Qiagen) according to the manufacturer's protocol. Total RNA was isolated from the frozen front paw tissue of hTNFtg and wild-type littermates by mechanical homogenization using a standard Trizol purification protocol.

### Quantitative real‐time polymerase chain reaction (qPCR)

RNA was reverse transcribed into cDNA using an Omniscript RT kit (Qiagen). RNA concentrations were determined using a Nanodrop spectrophotometer. qPCR was performed using a Fast Start SYBR Green I kit (Roche). The results were quantified by the 2−ΔΔC(t) method, using GAPDH expression levels for normalization. The primer sequences for human primers were as follows: *CXCL9* FW: ATCAGCACCAACCAAGGGACT, RV: GCTTTTTCTTTTGGCTGACCTG; *CXCL10* FW: ATTTGCTGCCTTATCTTTCTG, RV: TCTCACCCTTCTTTTTCATTGTAG; *CXCL11* FW: GAAGGATGAAAGGTGGGTGA, RV: AAGCACTTTGTAAACTCCGATG; *TNFSF13B*: FW: GGAGAAGGCAACTCCAGTCAGAAC, RV:CAATTCATCCCCAAAGACATGGAC; and *GAPDH* FW: TGATGACATCAAGAAGGTGGTGAAG, RV TCCTTGGAGGCCATGTGGGCCAT.

The following mouse primers were used: GAPDH FW TGGCATTGTGGA AGGGCTCATGAC, RV: ATGCCAGTGAGCTTGCCGTTCAGC; and *IRF1* FW: CCCACAGAAGAGCATAGCAC, RV: AGCAGTTCTTTGGGAATAGG.

### RNA sequencing

RNA was isolated as described above. The amount of total RNA was quantified using a Qubit Fluorometric Quantitation system (Thermo Fisher Scientific), and the RNA integrity number (RIN) was determined using the Experion Automated Electrophoresis System (Bio-Rad). RNA-seq libraries were prepared with a TruSeq Stranded mRNA LT sample preparation kit (Illumina) using both Sciclone and Zephyr liquid-handling robotics (PerkinElmer). Library concentrations were quantified with the Qubit Fluorometric Quantitation system (Thermo Fisher Scientific), and the size distribution was assessed using the Experion Automated Electrophoresis System (Bio-Rad). For sequencing, samples were diluted and pooled into NGS libraries in equimolar amounts. Expression-profiling libraries were sequenced with Illumina HiSeq 3000/4000 instruments in the 50-base-pair-single-end mode.

### RNA sequencing data analysis

Raw sequencing data were processed with Illumina2bam (http://github.com/wtsi-npg/illumina2bam) to generate unaligned BAM files. Sequence reads were mapped onto the human genome release hg38 (GRCh38) with Ensembl transcript annotation version 87 using tophat version 2.1.1^22^ with bowtie version 2.2.9^23^. Reads were counted with featureCounts^24^. Gene expression values (reads per kilobase exon per million mapped reads (RPKM)) were calculated with Cufflinks version 2.2^25^. The differential expression between two paired sample groups was calculated with edgeR^26^. The filtering for differentially expressed genes was performed with a *p*-value of 0.05 (FDR corrected) and minimal fold-change of 2. For GO enrichment analysis of a gene set, GOstats version 2.46.0^27^ was used. Cytoscape version 3.6.0 and the plugin Enrichment map (v3.0.0) were used for network visualization^28^.

### ELISA

A CXCL10 ELISA kit was purchased from Thermo Fisher Scientific. ELISAs were performed according to the manufacturer’s protocol.

### Statistical analysis

Unpaired and paired *t*-tests were used for comparing groups and paired samples, provided that the data exhibited a Gaussian distribution. For data that were not normally distributed, the Mann–Whitney *U* test or the Wilcoxon signed-rank test was performed. For graphing and statistical analysis, we used Graph Pad Prism 6 software.

## Results

### TNF-induced IRF1 expression in RA-FLSs

**Fig. 1:**
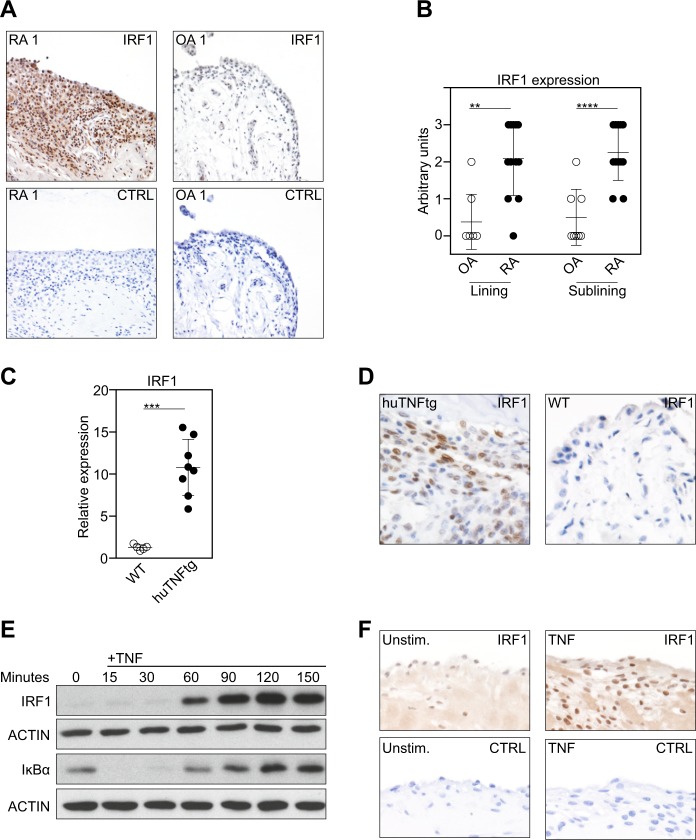
TNF-induced IRF1 expression in RA-FLSs. **a** Representative immunostaining for IRF1 (brown staining) in rheumatoid arthritis (RA) and osteoarthritis (OA) synovial tissue samples (upper panel). Staining with an isotype-matched control antibody (CTRL) is presented in the lower panel. **b** Synovial tissue samples from 12 RA and 8 OA patients evaluated for IRF1 expression using a semiquantitative score (0 = no staining, 3 = high staining). Lining: Mann–Whitney *U* test, **p = 0.0016; Sublining: Student’s *t*-test, ****p < 0.0001. **c** Quantitative RT-PCR analysis of the IRF1 mRNA levels in hind paws obtained from wild-type (WT) and hTNFtg mice. Mann-Whitney *U* test, ***p = 0.0008. **d** Immunohistochemical detection of IRF1 (brown staining) in hind paw tissue from WT and hTNFtg mice. **e** Western blot analysis of TNF-stimulated (10 ng/ml) RA-FLSs. Blots representative of at least five independent experiments with FLSs from different donors are shown. **f** RA-FLSs cultured in micromass organ cultures for 7 days in the presence or absence of TNF (10 ng/ml). Micromasses were fixed, sectioned, and stained with hematoxylin and a specific antibody against IRF1 (brown staining). Representative images from three independent experiments performed with FLSs from three RA patients are shown (upper panel). Staining with an isotype-matched control antibody (CTRL) is presented in the lower panel

In line with previous observations^5^, we found IRF1 to be highly expressed in the synovium in RA patients, but not in osteoarthritis (OA) patients (Fig. 1a) when we analyzed IRF1 expression by IHC. Further analyses revealed that IRF1 expression was distinctly elevated in fibroblast-like cells from both the synovial lining and sublining layers (Fig. 1b). Similarly, IRF1 was highly expressed in the inflamed synovium in human TNF transgenic (hTNFtg) mice (Fig. 1c, d) that constitutively overexpress human TNF, and consequently develop spontaneous arthritis. These data suggest a major role for TNF as a regulator of IRF1.

To confirm that TNF directs IRF1 expression in FLSs, we isolated FLSs from RA patients and stimulated these cells with TNF. We observed a strong upregulation of IRF1 expression upon TNF stimulation after 30 min, and this increase was maintained for up to 3 h (Fig. 1e). To address the effects of chronic TNF stimulation, we employed an in vitro 3-D synovial tissue culture model system, which was previously shown to display many in vivo functions of the synovial membrane^20^. To this end, RA-FLSs were cultured in a floating Matrigel matrix sphere and stimulated with TNF for 7 days. Similar to the expression pattern in RA synovial tissues (as shown in Fig. 1a), increased levels of IRF1 in the TNF-stimulated synovium-like tissue samples compared with unstimulated controls were revealed by IHC (Fig. 1f). These data define TNF as a regulator of IRF1 in RA-FLSs and provide an explanation for why IRF1 is abundantly expressed in the rheumatoid synovium, which is characterized by TNF overexpression^29^.

### The TNF-driven IFN response in FLSs depends on IRF1

**Fig. 2:**
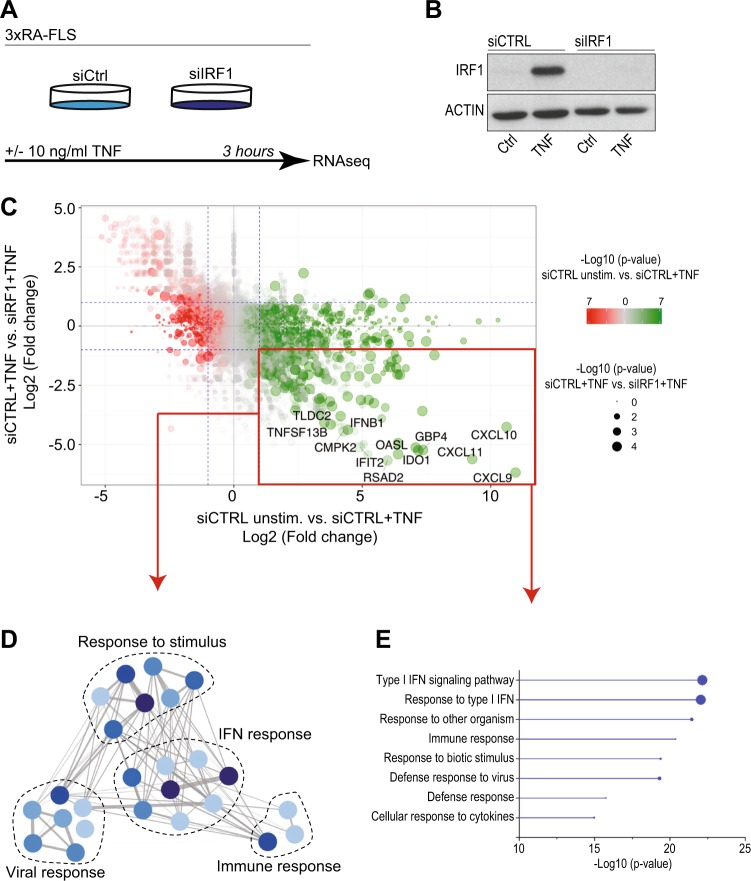
The TNF-driven interferon response in FLSs depends on IRF1. **a** Workflow outlining the RNA sequencing (RNA-seq) experiment. RA-FLSs from three different RA patients were transfected with nontargeting (siCTRL) or IRF1-targeting siRNA pools and stimulated with TNF (10 ng/ml) for 3 h. RNA was isolated and processed for transcriptomic profiling. **b** Representative immunoblots of IRF1 expression in RA-FLSs after siRNA transfection and stimulation with TNF (10 ng/ml) for 3 h. **c** Scatter-plot of the RNA-seq data (panel A) showing the impact of IRF1 knockdown on TNF-regulated genes. Dots in the lower right quadrant (red box) represent genes with expression upregulated by TNF, but impaired by the siRNA-mediated knockdown of IRF1 expression. Dashed blue lines indicate a twofold change in gene expression. **d** Network analysis of GO term (biological process, BP) enrichment among significantly regulated TNF-IRF1-dependent genes (as depicted in panel C, red box). The resulting network was calculated and visualized using EnrichmentMap. Groups of similar GO terms were manually circled. Line thickness is proportional to the similarity coefficient between the connected nodes. Node color is proportional to the FDR-adjusted *P*-value of the enrichment. **e** Enriched GO terms (BP, top 8) of the genes within the bottom right quadrant (red box) in the scatter-plot (Fig. 2c). Circle size shows the relative amount of significant genes associated with the GO term

To address the genome-wide contribution of IRF1 to the TNF-mediated response in RA-FLSs, we transfected RA-FLSs with siRNA pools targeting IRF1. The transfected RA-FLSs were stimulated with TNF for 3 h (Fig. 2a, b). Transcriptional changes were determined by RNA sequencing (Fig. 2, Supplementary Fig. 1). As expected, the comparison of TNF expression between stimulated and unstimulated cells revealed a strong upregulation of the expression of proinflammatory cytokines (e.g., *IL6* and *IL1B*), chemokines (*CXCL8*, *CXCL9*, *CXCL10*, and *CXCL11*), tissue-degrading enzymes (*MMP1* and *MMP10*) and genes (e.g., *VCAM*, *PTGS2*, and *TLR2*) that are associated with synovial inflammation. As highlighted in Fig. 2c, silencing IRF1 markedly diminished the expression of 121 TNF-upregulated genes, which revealed a genome-wide role for IRF1 as a transcriptional activator in FLSs. Most of these genes, such as the antiviral genes *RSAD2* and *IFIT2*; the proinflammatory CXCR3-binding chemokines *CXCL9*, *CXCL10*, and *CXCL11*; the B-cell activating factor *TNFSF13B* (BAFF) and *IFNβ*, represent IRGs.

Consistently, molecular function enrichment analyses of the 121 TNF-regulated, IRF1-dependent genes revealed an overrepresentation of pathways involved in IFN and viral responses (Fig. 2d, e). Overall, these results suggest that IRF1 is critical for the TNF-driven IFN response in FLSs.

### IRF1 is critical for the TNF-induced expression of CXCR3-binding chemokines and TNFSF13B

**Fig. 3:**
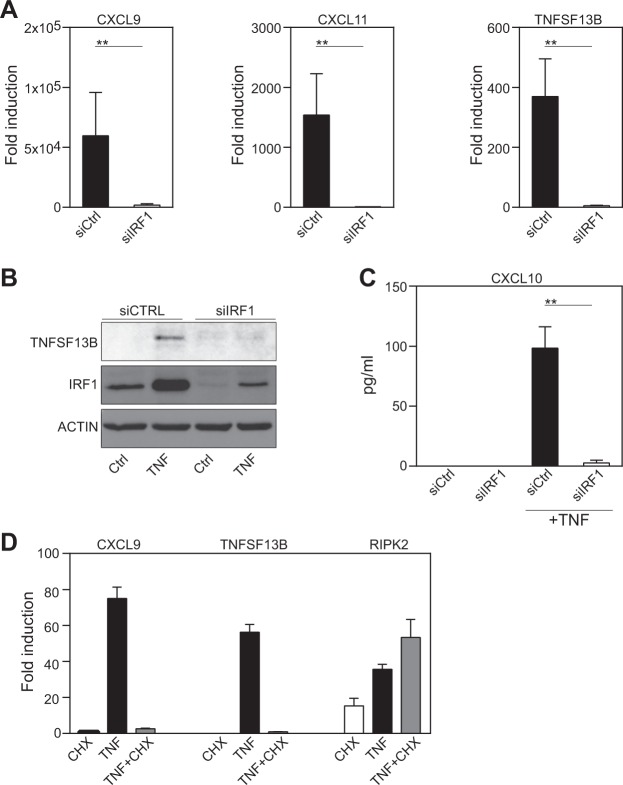
IRF1 is critical for the TNF-induced expression of CXCR3-binding chemokines and TNFSF13B. **a**–**c** FLSs were transfected with nontargeting (siCTRL) or IRF1-targeting siRNA pools. **a** Transfected FLSs from seven donors with RA were treated with TNF (10 ng/ml) for 6 h. Gene expression was determined by qPCR. Expression in the treated cells is presented relative to that in the unstimulated cells. Values are shown as the mean ± SEM. **p < 0.01, Wilcoxon matched-pairs test. **b** RA-FLSs were stimulated with TNF for 24 h. Representative western blots of at least four experiments with different RA-FLS cell lines are shown. **c**. IRF1 expression knockdown FLSs (*n* = 4) were treated with TNF (10 ng/ml) for 24 h. Supernatants were analyzed for CXCL10 expression by ELISA. Values are shown as the mean ± SEM. **d** qPCR was used to analyze gene expression in human RA-FLSs treated with TNF in the absence or presence of cycloheximide (CHX, 20 μg/ml). Each experiment was performed in technical triplicates (error bars, SEM of triplicates). Expression in the treated cells is presented relative to that in the unstimulated cells

To confirm the RNA sequencing data, we analyzed the expression of IFN-dependent chemokines and TNFSF13B. Consistent with the transcriptomic data, we observed diminished expression of CXCL9, CXCL10, CXCL11 and TNFSF13B in TNF-treated, IRF1-silenced FLSs on the mRNA level as assessed by qPCR (Fig. 3a) and on the protein level as shown by western blotting (Fig. 3b) or ELISA (Fig. 3c). To test whether the IRF1-induced regulation of IRGs upon TNF stimulation is dependent on de novo synthesis of IRF1, we exposed FLSs to cycloheximide (CHX) prior to TNF stimulation (Fig. 3d). Indeed, the addition of CHX, which blocks protein synthesis without affecting RNA transcription, completely abrogated the TNF-induced expression of *CXCL11* and *TNFSF13B*. As a control, we determined the expression of *RIPK2*, which is induced without new protein synthesis. As expected, *RIPK2* transcription was not affected by CHX treatment. These data support the importance of the TNF-driven de novo synthesis of IRF1 for the regulation of IRGs.

### The TNF-induced IFN response in FLSs is dependent on IFNβ

**Fig. 4:**
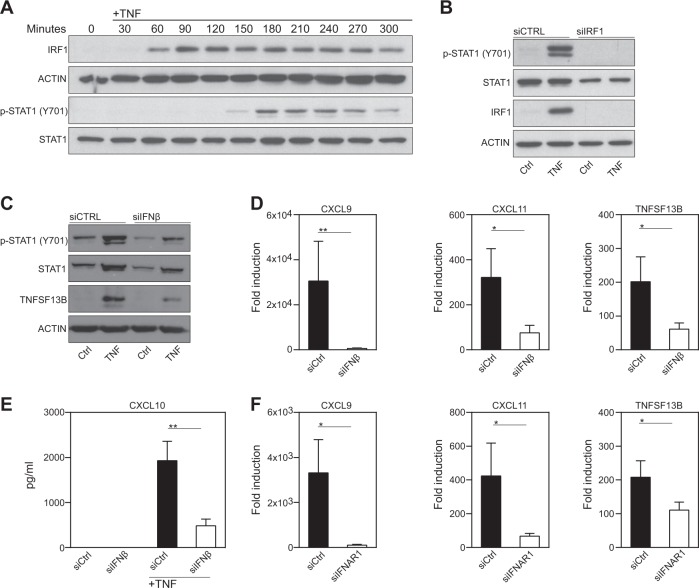
The TNF-induced interferon response in FLSs depends on IRF1. **a** Western blot analysis of IRF1 expression and STAT1 phosphorylation in TNF-stimulated (10 ng/ml) FLSs. Blots representative of three independent experiments with different RA-FLS cell lines are shown. **b** Western blots showing the IRF1 expression and STAT1 phosphorylation in TNF-treated (10 ng/ml; 3 h) RA-FLSs that were transfected with either nontargeting or IRF1-targeting siRNA pools. Blots representative of three independent experiments with FLSs from different RA donors are shown. **c**–**e** FLSs were transfected with nontargeting (siCTRL) or IFNβ-targeting siRNA pools and then stimulated with TNF (10 ng/ml). **c** Transfected FLSs stimulated with TNF for 24 h. Western blots representative of at least four experiments performed with different RA-FLS cell lines are shown. **d** Transfected FLSs from seven donors with RA treated with TNF (10 ng/ml) for 6 h. Gene expression was determined by qPCR. Expression in the treated cells is presented relative to that in the unstimulated cells. Values are shown as the mean ± SEM. *p < 0.05, **p < 0.01; Wilcoxon matched-pairs test. **e** Transfected RA-FLSs from seven different donors suffering from RA treated with TNF (10 ng/ml) for 24 h. Supernatants were analyzed for CXCL10 expression by ELISA. Values are shown as the mean ± SEM. **p < 0.01, Wilcoxon matched-pairs test. **f** FLSs (*n* = 6) transfected with nontargeting (siCTRL) or IFNAR1-targeting siRNA pools and then stimulated with TNF for 6 h. Gene expression was determined by qPCR. Expression in the treated cells is presented relative to that in the unstimulated cells. Values are shown as the mean ± SEM. *p < 0.05, Wilcoxon matched-pairs test. Western blots representative of at least four experiments with different RA-FLS cell lines are shown

TNF-induced, IFN-regulated gene expression in macrophages relies on the sequential activation of IRF1 and IFNβ, which induces the phosphorylation of the TF STAT1 upon binding to the type I IFN-receptor (IFNAR)^15^. We therefore performed time-kinetic analyses of IRF1 and p-STAT1 expression in TNF-stimulated FLSs. As shown in Fig. 4a, early expression of IRF1 was followed by the phosphorylation of STAT1. IRF1 knockdown by siRNA completely blocked STAT1 phosphorylation, which underlines the dependency of STAT1 phosphorylation on IRF1 expression (Fig. 4b). To explore whether IFNβ contributes to STAT1 activation and IRG expression in RA-FLSs, we silenced IFNβ with specific siRNA pools. Knocking down IFNβ expression was associated with decreased STAT1 activity (Fig. 4c). The RA-FLSs expressed lower amounts of TNFSF13B (Fig. 4c, d), *CXCL9*, CXCL10, and *CXCL11* in the absence of IFNβ (Fig. 4d, e). Similar results were obtained when IFN activity was blocked with an anti-IFNβ neutralizing antibody (Supplementary Fig. 2). STAT1 phosphorylation and IRG expression in the TNF-treated FLSs were also decreased when we targeted IFNAR by using either specific siRNA pools (IFNAR1, Fig. 4f and Supplementary Fig. 3) or a blocking antibody specific for IFNAR (Supplementary Fig. 4). These data suggest that the TNF-mediated upregulation of IRF1 expression induces the expression of IFNβ, which in turn activates the transcription factor STAT1 to induce the expression of IRGs in RA-FLSs.

### Baricitinib and tofacitinib inhibit the expression of CXCR3-binding chemokines and TNFSF13B

**Fig. 5:**
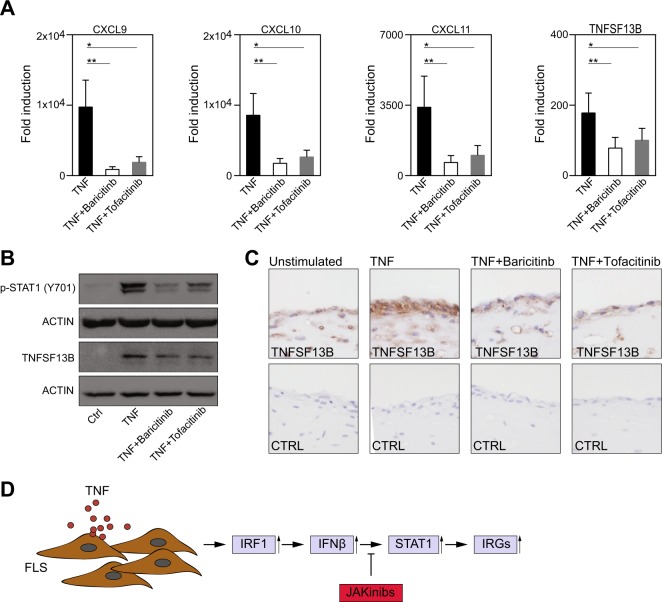
JAKinibs inhibit the expression of CXCR3-binding chemokines and TNFSF13B. **a** RA-FLSs (*n* = 7) were pretreated with DMSO, baricitinib (250 nM) or tofacitinib (250 nM) for 1 h and then stimulated with TNF (10 ng/ml) for 6 h. Gene expression was determined by qPCR. Expression in the treated cells is presented relative to that in the unstimulated cells. Values are shown as the mean ± SEM. *p < 0.05, **p < 0.01; Wilcoxon matched-pairs test. **b** RA-FLSs were pretreated with DMSO, baricitinib (250 nM) or tofacitinib (250 nM) for 1 h and then stimulated with TNF (10 ng/ml) for 24 h. Western blots representative of at least four experiments with different RA-FLS cell lines are shown. **c** RA-FLSs were cultured in micromass organ cultures for 8 days. After serum starvation overnight, the FLSs were treated with DMSO (Unstimulated), TNF (10 ng/ml) + DMSO, TNF (10 ng/ml) + baricitinib (250 nM) or TNF (10 ng/ml) + tofacitinib (250 nM) for 24 h. Micromasses were fixed, sectioned and stained with hematoxylin and a specific antibody against TNFSF13B (brown staining). Images representative of four independent experiments performed with FLSs from four different RA patients are shown. **d** The schematic representation of the TNF-induced pathway in FLSs is shown (JAKinib = Janus Kinase inhibitor; IRG = interferon-regulated gene)

The activation of STAT1 by IFNβ depends on the Janus kinases (JAKs) JAK1 and Tyk2^30^. Therefore, we tested whether JAKinibs, which are approved for the treatment of RA, can prevent the TNF-induced activation of STAT1 and the expression of downstream target genes. Indeed, both inhibitors diminished the expression of *CXCL9*, *CXCL10*, *CXCL11*, and *TNSF13B* in TNF-activated FLSs (Fig. 5a, b). Correspondingly, we observed decreased activation of STAT1 (Fig. 5b) in FLSs upon JAKinib treatment.

To explore the effects of JAKinibs on a tissue-like environment, we employed a 3-D synovial culture system. Tissue-bound FLSs exposed to TNF abundantly expressed TNFSF13B, as assessed by IHC. Both baricitinib and tofacitinib inhibited the expression of TNFSF13B, consistent with the results obtained from the 2-D cell cultures (Fig. 5c). Together these data support the conclusion that JAKinibs inhibit the TNF-induced, IRF1-mediated activation of the JAK-STAT pathway and the subsequent expression of IRGs (Fig. 5d) in RA-FLSs.

## Discussion

FLSs are increasingly recognized as major drivers of synovial inflammation, and joint destruction in RA^2,31^. Various proinflammatory cytokines can affect FLSs. Among proinflammatory cytokines, TNF strongly stimulates FLSs to produce cytokines and chemokines, which augment and perpetuate inflammatory cell recruitment and activation^32,33^. Thus, FLSs represent a promising target for the treatment of RA. However, compared with those in other cells, such as macrophages or lymphocytes, the pathways in FLSs that allow their participation in synovial inflammation are poorly defined. In this study, we showed increased IRF1 expression in inflamed human and mouse synovial tissue. Based on these data, we hypothesized that IRF1 is critical for the TNF response in FLSs. Transcriptomics, indeed, depicted IRF1 as a genome-wide activator of the TNF-induced expression signature in FLSs. Specifically, we found that several IRGs implicated in RA pathogenesis are controlled by IRF1. Increased levels of the CXCR3-binding chemokines (CXCL9, CXCL10, and CXCL11) can be found in the rheumatoid synovium. These chemokines are thought to sustain leukocyte recruitment to the inflamed synovial tissue. Moreover, CXCL10 has been shown to promote FLS invasiveness in an autocrine/paracrine manner^34^. Aside from chemokines, TNFSF13B was suppressed in the absence of IRF1. TNFSF13B, also known as BAFF, is abundantly expressed in RA synovial tissue. TNFSF13B is important for B-cell proliferation and differentiation as well as autoantibody production^35^. All these data underscore the importance of IRF1 as a key TF in synovial inflammation in RA.

Our biochemical studies revealed that IRF1 expression represents the first step of a temporally defined signaling circuit that almost exclusively controls the TNF-induced IFN response in FLSs. Knocking down IRF1 expression prevented the TNF-induced transcription of IFNβ and the subsequent activation of the TF STAT1. Consistently, both the lack of IFNβ and the loss of IFNAR function prevented the TNF-induced phosphorylation of STAT1. Our studies reveal a TNF-based signaling circuit that has also been observed in other cell types. In both macrophages and endothelial cells, TNF stimulation results in the expression of IRF1 and IFNβ to sustain the expression of proinflammatory chemokines via STAT1^14,15^. Thus, these observations suggest that this distinct pathway exists in the principal cellular components of the stromal tissue compartment. This conclusion is of particular interest, since stromal cells, such as fibroblasts, macrophages and endothelial cells, define the microenvironment in which inflammatory reactions take place. Especially in RA, activated stromal cells contribute to chronic inflammation and tissue damage by orchestrating the continuous recruitment, activation and retention of leukocytes^36^. Targeting the TNF-IRF1-IFNβ-STAT1 signaling circuit, which propels the expression of leukocyte-recruiting (e.g., CXCL9-CXCL10) and leukocyte-activating (e.g., TNFSF13B) inflammatory mediators, would therefore interrupt the persistent vicious crosstalk between immune cells and synovial stromal cells. These data support the concept of IFN-targeted therapies in RA patients. Ongoing phase 2 trials with anifrolumab are currently investigating the potential of type I IFN blockade in RA patients with a high interferon signature (NCT: 034356019). In macrophages, tofacitinib has been shown to suppress TNF-mediated STAT1 activation and chemokine expression^37^. Tofacitinib inhibits STAT1-driven CXCL10 expression in RA-FLSs^38^. In this study, we found that JAKinibs that are currently approved for the treatment of RA, namely, baricitinib and tofacitinib, suppress the expression of all three CXCR3-binding chemokines. Intriguingly, we found that the JAKinibs suppressed the expression of TNFSF13B, indicating that JAKinibs target B-cell-FLS crosstalk, which might therefore provide an additional mode of action. Our findings also provide evidence for a hitherto unrecognized quality of JAKinibs, namely, partial interference with TNF signaling pathways.

Together, these studies highlight the importance of the TNF-driven, IRF1-mediated regulation of the IFN pathway as a major contributor to FLS-mediated inflammation in RA. Considering the high number of nonresponders to disease-modifying antirheumatic drugs (DMARDS), a better understanding of the pathomechanisms and the informed use of biomarkers, such as the IFN signature, will ultimately lead to a more stratified treatment approach for RA patients.

## Supplementary information

Supplementary Table 1

Supplementary Figure 1

Supplementary Figure 2

Supplementary Figure 3

Supplementary Figure 4
