# Supplementary material for: IRF1 is critical for the TNF-driven interferon response in rheumatoid fibroblast-like synoviocytes: JAKinibs suppress the interferon response in RA-FLSs
Source: Exp Mol Med. 2019 Jul 8;51(7):75. doi: 10.1038/s12276-019-0267-6 (PMC6802656; doi:10.1038/s12276-019-0267-6)
Supplement: Supplementary file 1 — Supplementary Table 1 [file 12276_2019_267_MOESM1_ESM.pdf]

Supplementary Table 1.

	**RA** (n=12)	**OA** (n=8)
**Sex, female/male**	11/1	5/3
**Age, mean (range)**	53.3 (35-69)	73.4 (40-102)
**CDAI, mean (range)**	14.0 (2.3-36.3)	
**Prednislone, n**	11	
**Methotrexate, n**	7	
**Other csDMARD, n**	4	
**TNF-inhibitor, n**	2	

Supplementary Table 1.
Demographic and clinical characteristics of rheumatoid arthritis (RA) and osteoarthritis (OA) patients for immunohistochemical analyses.
