# Supplementary material for: IRF1 is critical for the TNF-driven interferon response in rheumatoid fibroblast-like synoviocytes: JAKinibs suppress the interferon response in RA-FLSs
Source: Exp Mol Med. 2019 Jul 8;51(7):75. doi: 10.1038/s12276-019-0267-6 (PMC6802656; doi:10.1038/s12276-019-0267-6)
Supplement: Supplementary file 2 — Supplementary Figure 1 [file 12276_2019_267_MOESM2_ESM.pdf]

Supplementary Figure 1.

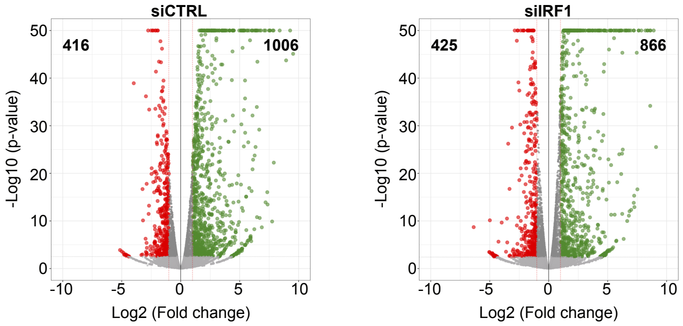

Supplementary Figure 1.
Vulcano blots showing the magnitude of differential expressed genes (unstimulated vs. TNF-stimulated). Bold numbers represent the amount of TNF-up (green) or down (red) regulated genes (fold change >2, FDR<0.05).
