# Supplementary material for: IRF1 is critical for the TNF-driven interferon response in rheumatoid fibroblast-like synoviocytes: JAKinibs suppress the interferon response in RA-FLSs
Source: Exp Mol Med. 2019 Jul 8;51(7):75. doi: 10.1038/s12276-019-0267-6 (PMC6802656; doi:10.1038/s12276-019-0267-6)
Supplement: Supplementary file 3 — Supplementary Figure 2 [file 12276_2019_267_MOESM3_ESM.pdf]

Supplementary Figure 2.

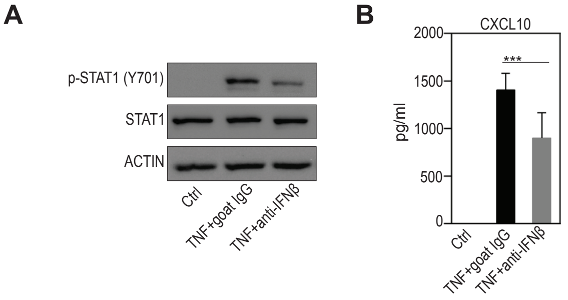

Supplementary Figure 2.
A. and B. FLS were stimulated with TNF for three (A.) or twentyfour hours (B.) in the presence of a neutralizing antibody to IFNβ (goat IgG, 50 μg/ml) or a non-specific goat IgG antibody (50 μg/ml).
A. Western blots of total and phosphorylated STAT1 expression in RA-FLS. Representative western blots of at least four experiments are shown.
B. Supernatants were analyzed for CXCL10 expression by ELISA. Values are the mean±SEM. n=8. Paired t-test. p=0.0008.
