# Supplementary material for: IRF1 is critical for the TNF-driven interferon response in rheumatoid fibroblast-like synoviocytes: JAKinibs suppress the interferon response in RA-FLSs
Source: Exp Mol Med. 2019 Jul 8;51(7):75. doi: 10.1038/s12276-019-0267-6 (PMC6802656; doi:10.1038/s12276-019-0267-6)
Supplement: Supplementary file 4 — Supplementary Figure 3 [file 12276_2019_267_MOESM4_ESM.pdf]

Supplementary Figure 3.

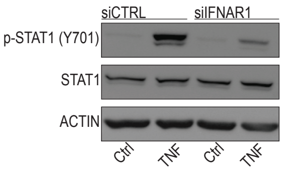

Supplementary Figure 3.
FLS were transfected with non-targeting (siCTRL) or IFNAR1-targeting siRNA pools. Transfected FLS were stimulated with TNF (10 ng/ml) for three hours. Western blots of total and phosphorylated STAT1 expression in RA-FLS.
