# Supplementary material for: IRF1 is critical for the TNF-driven interferon response in rheumatoid fibroblast-like synoviocytes: JAKinibs suppress the interferon response in RA-FLSs
Source: Exp Mol Med. 2019 Jul 8;51(7):75. doi: 10.1038/s12276-019-0267-6 (PMC6802656; doi:10.1038/s12276-019-0267-6)
Supplement: Supplementary file 5 — Supplementary Figure 4 [file 12276_2019_267_MOESM5_ESM.pdf]

Supplementary Figure 4.

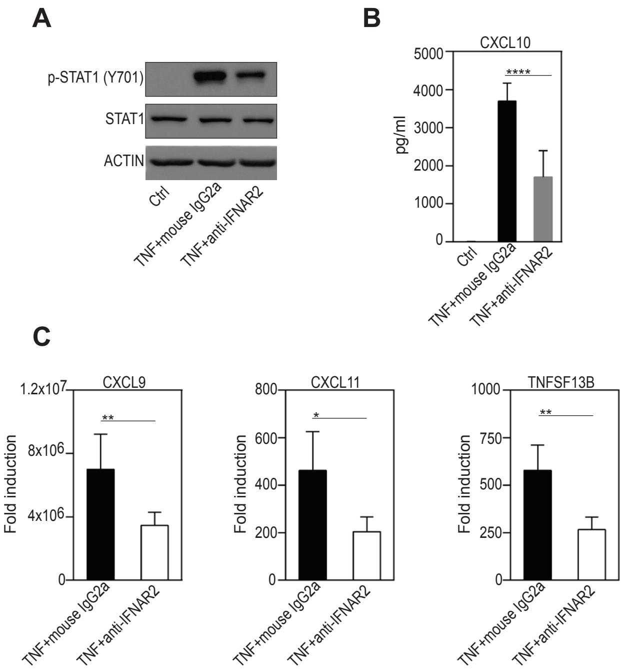

Supplementary Figure 4.
A. and B and C. FLS were stimulated with TNF for three (A.), twentyfour (B.) or six hours (C.) in the presence of an anti-human IFNAR2 (mouse IgG2a, clone MMHAR-2, 4 µg/ml) blocking or a non-specific mouse IgG2a antibody (4 µg/ml).
A. Western blots of total and phosphorylated STAT1 expression in RA-FLS. Representative western blots of at least four experiments are shown.
B. Supernatants were analyzed for CXCL10 expression by ELISA.Values are the mean±SEM. n=8. Paired t-test. $p<0.0001$.
C. qPCR analysis of CXCL9 (Wilcoxon matched pairs test, $p<0.005$), CXCL11 (Paired t-test, $p<0.02$) and TNFSF13B (Paired t-test, $p<0.005$) expression in RA-FLS. Expression is presented relative to unstimulated cells. Values are the mean± SEM. n=8.
